# Inheritance and phylogenetic analysis of *Tylosurus acus melanotus* based on the complete mitochondrial genomes

**DOI:** 10.1080/23802359.2016.1180551

**Published:** 2016-07-10

**Authors:** Hong Zhang, Da Zeng, Youhou Xu, Yanqiu Zhang, Huaiyi Fang, Lili Xu, Bin Gong

**Affiliations:** Guangxi Key Laboratory of Beibu Gulf Marine Biodiversity Conservation, Guangxi Colleges and Universities Key Laboratory of Exploitation and Protection of Beibu Gulf Marine Biological Resources, Qinzhou University, Guangxi, China

**Keywords:** Gene arrangement, inheritance and evolutionary relationship, mitogenome, *Tylosurus acus melanotus*

## Abstract

In our research, 17 sets of primers were used to amplify contiguous, overlapping segments of the complete mitochondrial DNA (mtDNA) of the *Tylosurus acus melanotus* in order to analyse the inheritance and evolutionary relationship of this species. The total length of the mitochondrial genome is 16,723 bp and deposited in the GenBank with accession numbers KU605633. The gene arrangement was similar to other bony fishes which contained 37 genes (13 protein-coding genes, 2 ribosomal RNA and 22 transfer RNAs) and a major non-coding control region. Most genes were encoded on the H-strand, except for the ND6 and 8 tRNA genes, encoding on the L-strand. The mtDNA may provide some important genetic background information for this valuable fish. The nucleotide skewness for the coding strands of *T. acus melanotus* (GC-skew = −0.21) is biased towards G and the negative GC-skew ranges from -0.53(ATP8) to -0.12(CO2) and the AT-Skew showed more positive varying from −0.17(ND3) to 0.26(ATP6). The phylogenetic analysis demonstrated that *T. acus melanotus* was clustered together with *Ablennes hians*, but far away from *Cololabis saira* which belongs to Beloniformes, Belonidae.

Mitochondria are double-layered membrane-bound organelles with some unique characteristics, including maternal inheritance, small size (16K), lack of recombination and high evolutionary rate (Chen et al. 2012). The mitogenome has been widely considered as a natural bio-marker for population genetic and evolutionary studies (Su et al. 2014). For a better understanding of inheritance and evolutionary relationship, we focused on the information of the complete mitochondrial genomes from *Tylosurus acus melanotus*.

In the present study, the sample was collected from the South China Sea (37.12°E, 118.28°N). The dorsal myotome of the fish was obtained and preserved in 95% ethanol with the DNA isolation Kit (QIAGEN, USA) to obtain the genomic DNA. The genomic DNA was stored in −20 °C. The total length of the mitochondrial genome is 16,723 bp and deposited in the GenBank with accession number KU605633. The gene arrangement and genome size of the mitochondrial genomes were similar to other bony fishes (Chen et al. 2012; Li et al. 2010). The mitochondrial DNA (mtDNA) was composed of 13 protein-coding genes, 2 ribosomal RNA (rRNA) genes, 22 transfer RNA (tRNA) genes and 1 control region (D-loop). In the mitochondrial genome, abundant genes were derived from the H-strand, whereas only ND6 and 8 tRNA genes were derived from the L-strand. In total, 13 protein-coding genes had ATG as start codon except CO1 which had GTG as start codon.

However, the termination codons of the 13 protein-coding genes are varied because they have TAA, TA– and T–– as stop codons, which are inconsistent with other bony fishes that have TAA, TA–, T– and TAG as stop codons. Except for tRNA-Ser (AGY), the other 22 tRNA genes could be formed into a typical cloverleaf structure without dihydrouridine arm. With an estrangement genetic affinity, the 22 tRNA genes were observed with IQM (tRNA-Ile, tRNA-Glu and tRNA-Met), WANCY (tRNA-Trp, tRNA-Ala, tRNA-Asp, tRNA-Cys and tRNA-Tyr) and HSL (tRNA-His, tRNA-Ser and tRNA-Leu) and other 11 single tRNA.

As observed in other bony fish, the G contents were lowest (17.13%) (Oh et al. 2007). Similar to previous report, AT-Skew and GC-skews usually show high levels of variation, indicating the effects of phylogenetic analyses (Nesnidal et al. 2011). The nucleotide skewness for the coding strands of *T. acus melanotus* (GC-skew = −0.21, AT-skew =0.02) is biased towards A and C. A similar trend has been observed in other teleost mitogenomes: the negative GC-skew ranges from −0.53(ATP8) to −0.12(CO2) and the AT-skew showed more positive varying from −0.17(ND3) to 0.26(ATP6).

Phylogenetic analysis was performed using the mitochondrial genomes of other fish species (Figure 1). Each of the datasets was aligned using Clustal X and analysed by Maximum parsimony (M-P) in MEGA 4.0 and bootstrap analysis was performed with 1000 replications (Tamura et al. 2007). The result demonstrated that the *T. acus melanotus* was clustered together with *Ablennes hians.* However, the *T. acus melanotus* was clustered far away from *Cololabis saira* which belongs to Beloniformes, Belonidae.

**Figure 1.**
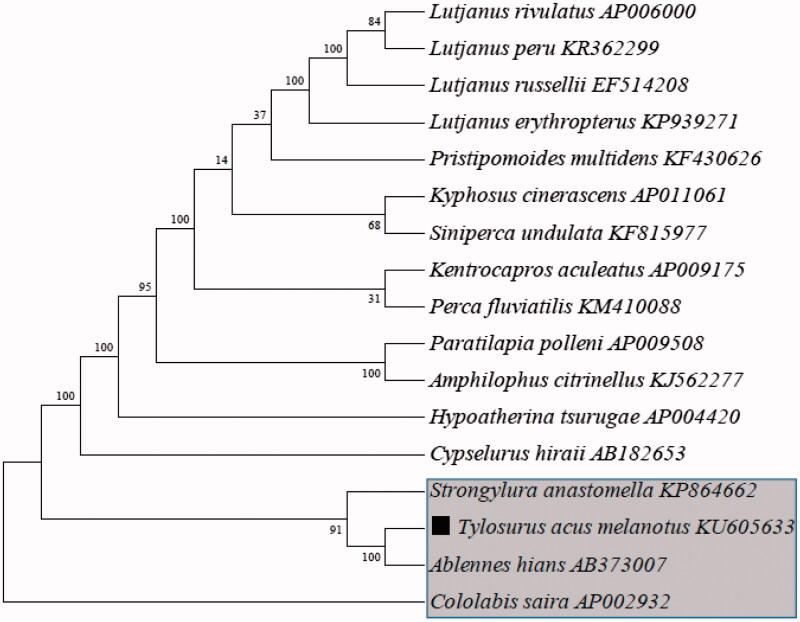
Phylogenetic analysis based on mitochondrial genome sequences with maximum likelihood distance (M-P) method.
